# Chemosensory Contributions of E-Cigarette Additives on Nicotine Use

**DOI:** 10.3389/fnins.2022.893587

**Published:** 2022-07-19

**Authors:** Natalie L. Johnson, Theresa Patten, Minghong Ma, Mariella De Biasi, Daniel W. Wesson

**Affiliations:** ^1^Department of Pharmacology and Therapeutics, Center for Smell and Taste, Center for Addiction Research and Education, University of Florida, Gainesville, FL, United States; ^2^Department of Psychiatry, Perelman School of Medicine, University of Pennsylvania, Philadelphia, PA, United States; ^3^Pharmacology Graduate Group, Perelman School of Medicine, University of Pennsylvania, Philadelphia, PA, United States; ^4^Department of Neuroscience, Perelman School of Medicine, University of Pennsylvania, Philadelphia, PA, United States

**Keywords:** flavors, TRPA1, TRPM8, addiction, menthol, olfactory, trigeminal, gustatory

## Abstract

While rates of smoking combustible cigarettes in the United States have trended down in recent years, use of electronic cigarettes (e-cigarettes) has dramatically increased, especially among adolescents. The vast majority of e-cigarette users consume “flavored” products that contain a variety of chemosensory-rich additives, and recent literature suggests that these additives have led to the current “teen vaping epidemic.” This review, covering research from both human and rodent models, provides a comprehensive overview of the sensory implications of e-cigarette additives and what is currently known about their impact on nicotine use. In doing so, we specifically address the oronasal sensory contributions of e-cigarette additives. Finally, we summarize the existing gaps in the field and highlight future directions needed to better understand the powerful influence of these additives on nicotine use.

## It Is Important to Consider the Sensory Aspects of E-Cigarette Use

The United States (US) is currently experiencing what has been termed a “teen vaping epidemic,” as adolescent use of electronic cigarettes (e-cigarettes) has dramatically increased in recent years (Centers for Disease Control and Prevention [CDC], 2020; Huey et al., 2020; Printz, 2020). From 2017 to 2018, e-cigarette use, commonly referred to as vaping, among high school students almost doubled from 11.7 to 20.8% (Centers for Disease Control and Prevention [CDC], 2019). E-cigarettes are marketed as smoking cessation aids, and while there may be some merit to these claims (Hartmann-Boyce et al., 2021; Zhang et al., 2021), other studies disagree (Chen et al., 2020) and even point to e-cigarettes as a cause of relapse in former smokers (Baenziger et al., 2021). Furthermore, many young e-cigarette users are “nicotine-naïve,” having never smoked nor used tobacco products before initiating e-cigarette use. Nicotine use, especially during adolescence, results in short- and long-term detriments to brain development (Slotkin, 2004; Yuan et al., 2015; Leslie, 2020), reward processing (Mansvelder et al., 2002; Hilario et al., 2012; Ren and Lotfipour, 2019), and overall health (Picciotto et al., 2002; Lee and Cooke, 2011; Kutlu et al., 2015). Several studies have also found that initiation of e-cigarette use in adolescence or adulthood increases the likelihood of later combustible cigarette use (Barrington-Trimis et al., 2016; Berry et al., 2019; Baenziger et al., 2021; Hair et al., 2021; Zhang et al., 2021), which is alarming considering that smoking remains the leading cause of preventable death and disease in the United States (Centers for Disease Control and Prevention [CDC], 2020). The rapid rise in e-cigarette popularity and subsequent nicotine use, especially among youth, has sparked a public health concern.

E-cigarettes are often associated with thousands of available “flavors,” and the vast majority of e-cigarette users vape flavored products (Landry et al., 2019). Perhaps unsurprisingly, users point to these attractive flavors (*e.g.*, tiramisu, watermelon apple, and cinnamon funnel cake) as a primary reason for e-cigarette experimentation (Kong et al., 2015; Baker et al., 2021b). Flavored e-cigarette users also have a lower intention rate of quitting e-cigarette or tobacco product use (Dai and Hao, 2016). The “characterizing flavors” in e-cigarette products are defined as a “clearly noticeable smell or taste other than one of tobacco, resulting from an additive or combination of additives, including, but not limited to, fruit, spice, herb, alcohol, or candy which is noticeable before or during the consumption of the tobacco product” (Talhout et al., 2016). This review will focus on the sensory aspects associated with e-cigarette use and the contributions of characterizing flavors to nicotine consumption.

## Flavor Additives in Combustible Cigarettes and a Transition to Electronic Cigarettes

Internal tobacco company documents indicate that top companies such as Phillip Morris and RJ Reynolds invested exorbitant sums of money along with years of research into the effects of flavor additives on consumer preferences and use. For example, sugars, cocoa, licorice, and ammonium were all added based on their presumed ability to improve taste, reduce bitterness, and facilitate nicotine uptake (Rabinoff et al., 2007). Furthermore, tobacco company research found that teenagers especially were more curious and susceptible to fruity and sweet flavored products, and therefore big tobacco focused their marketing strategies accordingly (Carpenter et al., 2005). As a testament to their marketing success, surveys conducted in 2004 found that a far greater proportion of young adult smokers aged 17–19 used flavored cigarettes compared to those over 25 (Lewis and Wackowski, 2006). That same year, news outlets such as The Wall Street Journal described sweet-flavored cigarettes as “one of the hottest new product categories in the tobacco industry” (O’Connell, 2004). Eventually, in 2009, as part of the continued effort to limit youth smoking, the US Food and Drug Administration (FDA) passed the Family Smoking Prevention and Tobacco Control Act which banned characterizing flavors, except for menthol, from combustible cigarettes.

Amidst the general movement toward abolishing cigarette smoking, tobacco companies and independent entities alike worked to develop new nicotine delivery systems that could exist in the changing environment. Tobacco companies had numerous projects dating back to the 1960s with the goal of developing more “socially acceptable” cigarettes with less visible smoke or odor (Ling and Glantz, 2005). However, the first e-cigarette to hit the market was a device patented and released in China in 2004 by pharmacist Hon Lik. It was the first of its kind to heat and aerosolize nicotine-containing liquid solutions, e-liquids, for user inhalation. First-generation devices, such as Lik’s “Ruyan,” looked similar to traditional cigarettes (“cig-a-likes”) and were constructed as single, disposable units. E-cigarettes reached the European and US markets around 2007 and since their initial introduction, they have quickly evolved. While the basic mechanics remain the same, e-cigarettes now widely vary in size, operating specifications, liquid storage, and overall liquid composition. Unifying features among e-cigarettes are the presence of nicotine, propylene glycol and vegetable glycerin as e-liquid solvents, and characterizing flavors.

## Sensory Experience of Smoking and Vaping

The sensory aspect of smoking plays a pivotal, yet often overlooked, role in nicotine use. As a notable example, smokers report greater satisfaction from smoking a denicotinized cigarette over intravenous nicotine delivery, thus emphasizing how airway irritation associated with smoking is crucial to the overall experience (Rose, 2006). As previously discussed, tobacco companies relied heavily on chemosensory research, and industry researchers concluded that flavor additives helped alleviate harshness and improve taste, ultimately enhancing the sale and use of their products (for review, see Carpenter et al., 2007).

While examining the sensory impact of flavor additives on e-cigarette use in this review, we find it helpful to first consider the other base components of an e-liquid (*i.e.*, nicotine, propylene glycol, and vegetable glycerin) that are ingested by the user and their sensory contributions. We will then move to a discussion of characterizing flavors, starting with menthol, wherein we examine current literature across both rodent and human studies and the role of sensory perception on overall e-cigarette use.

## Nicotine

**FIGURE 1:**
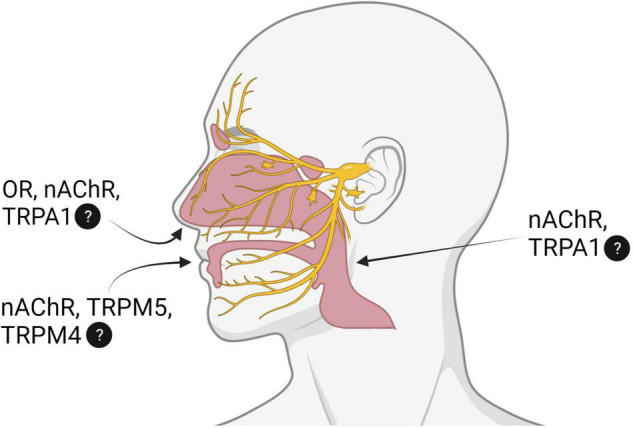
Simplified schematic depicting the receptor mechanisms responsible for nicotine perception in the nose, mouth, and throat. OR, olfactory receptor; nAChR, nicotinic acetylcholine receptor; TRPA1, TRPM4, TRPM5, transient receptor potential (TRP) ankyrin 1, melastatin 4, melastatin 5. Question marks indicate current gaps or debate within the field concerning nicotine’s actions on TRPM4, TRPA1. Additional specific details can be found in Table 1. Created with BioRender.com.

Nicotine is the primary psychoactive ingredient responsible for the rewarding and reinforcing effects of tobacco products. Nicotine’s actions, including some of its sensory effects, are mediated through nicotinic acetylcholine receptors (nAChRs) expressed throughout the central and peripheral nervous systems. Detailed reviews on the effects of nicotine mediated by these systems can be found elsewhere (De Biasi and Dani, 2011; Papke et al., 2020). Here we will discuss the sensory effects of nicotine and the receptor mechanisms that may mediate nicotine perception in the mouth, nose, and throat. As illustrated in Figure 1, along with nAChRs, nicotine may act upon olfactory receptors (ORs) and transient receptor potential (TRP) ankyrin 1, melastatin 4, and melastatin 5 (TRPA1, TRPM4, and TRPM5). We will also discuss the impact of sensory input on tobacco use, implications of pH, and possible effects on e-cigarette use (see Table 1).

**TABLE 1:** Summary of nicotine’s sensory effects.

Sensory modality	Description	Mechanism/receptor target	Impact on nicotine use	References
Taste	Bitter	Bitter taste receptor type 2 (T2R), TRPM5, TRPM4	• Individuals with higher thresholds for bitter compounds PTC and PROP more likely to be heavy smokers	Enoch et al., 2001; Cannon et al., 2005; Snedecor et al., 2006; Mangold et al., 2008
Odor	Sweet, warm, and spicy	Olfactory sensory neurons (OSNs)	• Smokers rate nicotine odor as more pleasant than non-smokers • Odor of cigarette smoke heightens nicotine craving in adult smokers • E-cigarette users report the smell of their vape as pleasant • Pinching the nose while vaping to block the olfactory percept of volatiles reduces perceived sweetness and intensity	Hummel et al., 1992; Thuerauf et al., 2000; Cortese et al., 2015; Rosbrook et al., 2017; DiPiazza et al., 2020
Touch/pain	Irritation, burning, “scratchy”	Nicotinic acetylcholine receptors (nAChRs)	• Smokers report greater enjoyment when smoking denicotinized cigarette over intravenous nicotine delivery • Anesthetizing airway *via* gurgling/inhaling lidocaine prior to smoking decreases craving and desirability of cigarettes • Inhaling respiratory irritants such as citric acid or black pepper reduces nicotine cravings and promotes smoking abstinence • Irritation felt at the back of the throat while vaping (*i.e.*, throat hit) reported as a desired quality in experienced users	Rose et al., 1984; Rose and Behm, 1994; Westman et al., 1995; Rose, 2006; Pokhrel et al., 2015; Cohn et al., 2020

*TRPM4/5, transient receptor potential melastatin 4/5; PTC, phenylthiocarbamide; PROP, 6-n-propylthiouracil.*

### Nicotine Taste

Upon oral ingestion, nicotine activates gustatory pathways and is generally described as tasting bitter (Iwasaki and Sato, 1981; Dahl et al., 1997). Bitter taste is mediated through the taste receptor type 2 (T2R) family of G-protein coupled receptors that are expressed by taste receptor cells of the tongue and palate epithelium (Andres-Barquin and Conte, 2004). Bitter, along with umami and sweet taste, rely on different receptor systems, but utilize a common transduction pathway, reliant on the signaling effector, phospholipase C (PLC), and the calcium-sensitive TRPM5 (Zhang et al., 2003). Upon oral ingestion, nicotine can act through a TRPM5-dependent pathway, similar to other bitter tastants such as quinine (Oliveira-Maia et al., 2009). However, while TRPM5 knockout (KO) mice show no oral aversion to quinine, these mice maintain some aversion to nicotine (Oliveira-Maia et al., 2009). Furthermore, recordings of the chorda tympani (CT) nerve from TRPM5 KOs show reduced, yet present, responses to nicotine (Oliveira-Maia et al., 2009), indicating that nicotine taste transduction also relies on a TRPM5-independent pathway.

Lingual application of the nAChR antagonist hexamethonium significantly reduces CT nerve responses to nicotine, implicating nAChRs in nicotine taste (Schiffman, 2000). Additionally, nicotine’s activation of gustatory neurons in the nucleus of the solitary tract (NTS) is blunted by lingual application of the nAChR antagonist, mecamylamine (Simons et al., 2006). This effect appears specific to gustatory pathways since trigeminal ganglionectomy does not affect the nicotine-evoked response of NTS neurons (Simons et al., 2006). In support of a TRPM5-independent and nicotine-specific taste response, Oliveira-Maia et al. (2009) found that nAChRs are expressed in both taste receptor cells and the CT nerve and play a role in nicotine taste transduction. Application of mecamylamine significantly reduced CT responses to nicotine while having no effect on responses to other bitter, sweet, or salty tastants. Furthermore, in rats, discrimination between quinine and nicotine in a two alternative choice task was impaired when nicotine was presented simultaneously with mecamylamine. Importantly, even firing activity of gustatory cortex neurons discriminated between bitter stimuli (Oliveira-Maia et al., 2009). Together this work implicates both TRPM5 and nAChRs in nicotine taste.

Recent work has implicated TRPM4 as another critical player in bitter, sweet, and umami taste signaling (Banik et al., 2018). TRPM4 is likely relevant for nicotine taste, as slight CT responses remain in mecamylamine-treated TRPM5-KO mice (Oliveira-Maia et al., 2009); however, this relationship has not been examined directly.

### Taste and Nicotine Use

Individuals with a high taste threshold for bitter compounds including phenylthiocarbamide (PTC) and propylthiouracil (PROP), also known as “non-tasters”, are more likely to be heavy smokers (Enoch et al., 2001; Cannon et al., 2005; Snedecor et al., 2006; Mangold et al., 2008). Taste sensitivity to PTC and PROP, a heritable trait in humans, is influenced by polymorphisms in bitter taste receptor genes (Guo and Reed, 2001). Nicotine is the primary source of bitter taste in e-cigarettes, and there is a positive association between nicotine concentration and ratings of bitterness (Pullicin et al., 2020). While the relationship between taster status and e-cigarette use has not been directly examined, it is interesting to consider whether non-tasters of PTC/PROP are also more prone to e-cigarette use.

### Nicotine Odor

Although nicotine can exist in one of two isomers, (S)-nicotine and (R)-nicotine, nicotine derived from tobacco is almost entirely represented (>99%) by (S)-nicotine (Armstrong et al., 1998). Synthetic nicotine is an emerging concern since tobacco-free nicotine (TFN) products are becoming more prevalent. TFN products are often marketed as cleaner, purer, toxin-free alternatives to their tobacco-derived counterparts (Jordt, 2021). Levels of (R)-nicotine vary across TFN products but can be as high as 50% (Hellinghausen et al., 2017). Therefore, it is worthwhile to discuss the sensory implications of both (S)- and (R)-nicotine. While little is known about the gustatory perception of nicotine isomers, several studies have examined the olfactory and trigeminal perception of (S)- and (R)-nicotine.

Nicotine is described as having a sweet, warm, or spicy odor at low concentrations. Since mecamylamine administration does not affect detection threshold or rated intensity of nicotine odor, olfactory perception of nicotine appears to be independent of nAChRs (Thuerauf et al., 2005). Recordings from cultured rat and human olfactory sensory neurons (OSNs) agree with this finding, as nAChR antagonists mecamylamine and hexamethonium do not impact OSN responses to nicotine (Bryant et al., 2010). Instead, nicotine binds to ORs expressed by OSNs and triggers a canonical olfactory signaling pathway, dependent on activation of adenylyl cyclase and calcium influx (Bryant et al., 2010). Some isomer specificity is observed in the rat olfactory system, as OSNs respond to either (S)-nicotine or (R)-nicotine; however, the vast majority (∼96%) of human OSNs are not stereoselective (Bryant et al., 2010). This lack of stereoselectivity in human OSNs seems at odds with studies which have consistently reported that humans can discriminate between nicotine isomers (Hummel and Kobal, 1992; Thuerauf et al., 2000). A smaller population of selective OSNs may exist that mediate this distinction. Alternatively, trigeminal influences may play a role in discrimination. Indeed, nicotine, even when presented at low, “olfactory concentrations” that do not induce perceptible burning or stinging sensations, activates human trigeminal cortical areas (Albrecht et al., 2009). Furthermore, the threshold by which (S)- and (R)-nicotine evoke burning and stinging sensations vary, with individuals having higher thresholds for (R)-nicotine (Thuerauf et al., 1999). Therefore, even at low nicotine concentrations, trigeminal influences may still aid in perception.

### Olfaction and Nicotine Use

As briefly mentioned, smokers and non-smokers alike can discriminate between the odors of (S)- and (R)- nicotine (Hummel et al., 1992; Thuerauf et al., 2000). Hummel et al. found that both smokers and non-smokers rate the odor of (R)-nicotine as unpleasant, yet only smokers rate (S)-nicotine as pleasant. However, Thuerauf et al. found that smokers rate both (R)- and (S)-nicotine odor as more pleasant than non-smokers. Differences in odor delivery likely account for the variation between studies. Hummel et al. presented nicotine odors by squeezing bottles near the participant’s nose while Thuerauf delivered odors using an olfactometer with nitrogen as their carrier gas to control for possible effects of oxidation and degradation on nicotine’s odor. Both studies agree, however, in that smokers find (S)-nicotine odor more pleasant than non-smokers, emphasizing the role of experience in hedonic ratings of nicotine’s sensory cues. Odors associated with cigarette and e-cigarette use likely become conditioned cues predictive of nicotine reward. For example, the odor of cigarette smoke, comprised of over 400 odorants which activate an array of odorant receptors (McClintock et al., 2020), heightens nicotine cravings in adult smokers (Cortese et al., 2015). In adult e-cigarette users, 99% of study participants reported a feeling of pleasantness while vaping, and 88% of them identified smell as a contributing factor (DiPiazza et al., 2020). 85% of users also rated the smell of someone else vaping as pleasant (DiPiazza et al., 2020).

Beyond their possible role as a conditioned cue, odors also impact taste perception (Grant, 1963; Verhagen and Engelen, 2006; Isogai and Wise, 2016). In humans, odors are perceived either orthonasally or retronasally. Orthonasal olfaction refers to the perception of smell triggered when odors are inhaled into the nose. In contrast, retronasal olfaction arises during eating and drinking when odor volatiles are released in the mouth and enter the nasal cavity through the nasopharynx as air is driven out of the nostrils. The effect of odors on taste perception is largely attributed to retronasal olfaction, which is likely integral to e-cigarettes as aerosols are inhaled *via* the mouth. For example, simultaneous oral delivery of bitter tastants with a sweet, “fruity,” aromatic flavorant, ethyl hexanoate, decreases perceived bitterness (Isogai and Wise, 2016). However, pinching the nose to block the orthonasal percept of volatiles while vaping reduces perceived sweetness and intensity (Rosbrook et al., 2017). As will be discussed in a later section, sweetness ratings are positively associated with e-cigarette liking. Further, individuals rate odor-rich liquids similarly in terms of liking and intensity when smelled or vaped (Krüsemann et al., 2020). Together these studies emphasize the importance of flavor volatiles and olfaction, both when experienced ortho- and retronasally, in the sensory evaluation of e-cigarette and nicotine use.

### Nicotine as an Irritant

Nicotine inhalation *via* smoking or vaping commonly results in coughing and irritation of the throat, mouth, and nose, especially in naïve users. When applied to the tongue or nasal cavity, nicotine causes pain and discomfort, which is reduced by the application of nAChRs antagonists (Jarvik and Assil, 1988; Carstens et al., 1998; Dessirier et al., 1998; Alimohammadi and Silver, 2000; Thuerauf et al., 2005), indicating that nicotine’s irritant effects in the mouth and nose are predominately mediated by nAChRs located on trigeminal nerve endings (Wang et al., 1993; Alimohammadi and Silver, 2000; Keiger et al., 2003). Airway and lung irritation also depends on nAChRs expressed in vagal afferent fibers (Xu et al., 2007; Gu et al., 2008; Chung and Widdicombe, 2009; Lee L. Y. et al., 2018).

(S)-nicotine activates TRPA1 channels in cultured mouse trigeminal ganglion neurons and when human TRPA1 is heterologously expressed (Talavera et al., 2009). Similarly, utilizing heterologously expressed human TRPA1 receptors and cultured trigeminal ganglion neurons, Schreiner et al. (2014) found that (R)-nicotine also activates both human TRPA1 channels and trigeminal ganglion neurons. They also provide evidence of another unknown receptor expressed in cultured trigeminal ganglion neurons that is sensitive to (S)-nicotine, since nAChR and TRPA1 antagonists failed to completely abolish nicotine-induced responses (Schreiner et al., 2014). However, the involvement of TRPA1 in nicotine irritation is not clear-cut. Zhang et al. (2015) found in both mice and rats that only application of mecamylamine, but not the selective TRPA1 antagonist HC-030031, inhibited nicotine’s activation of dorsal root ganglion neurons (Albers et al., 2014). Similarly, a study conducted in guinea pigs reported that HC-030031 did not affect nicotine-induced bronchoconstriction while mecamylamine prevented it (Lee L. Y. et al., 2018). Furthermore, HC-030031 had no effect on nicotine-induced currents in cultured human dorsal root ganglion neurons that were blocked by mecamylamine (Zhang et al., 2019). Together these studies emphasize nAChRs as the primary mediator of nicotine’s irritant effects and not TRPA1. Species differences may account for discrepancies among studies (Zhang et al., 2019). However, methodological differences such as nicotine’s concentration and pH, application times, and *in vitro* versus *in vivo* preparations are also likely factors (Kichko et al., 2013; Zhang et al., 2015). Taken together, the precise role of TRPA1 in nicotine irritation is in need of more definition.

### Irritation and Nicotine Use

The irritating sensation felt at the back of the throat while vaping, also known as “throat hit,” is a quality that some e-cigarette users report liking (Pokhrel et al., 2015; Cohn et al., 2020). Nicotine’s sensory effects on the airway, mediated through nAChRs and possibly TRP channels, are integral to overall smoking enjoyment. As previously mentioned, smokers report greater enjoyment when smoking a denicotinized cigarette over intravenous nicotine delivery (Rose, 2006). Along with this, anesthetizing the airway *via* gurgling or inhaling lidocaine just prior to smoking decreases craving and desirability of cigarettes (Rose et al., 1984). Furthermore, inhaling respiratory irritants such as citric acid or black pepper reduces nicotine cravings and promotes smoking abstinence (Rose and Behm, 1994; Westman et al., 1995). Nicotine’s involvement in throat hit is supported by a wide-scale, exploratory study reporting a relationship between increasing nicotine concentration and throat hit (Etter, 2016). Interestingly, however, other studies have found that while increasing nicotine concentrations does increase throat hit, this does not increase overall appeal (Goldenson et al., 2016). Therefore, while some degree of airway irritation is often desired, greater throat hit does not always correlate with a more positive experience. User experience likely impacts desired sensory stimulation, as dual cigarette and e-cigarette users or individuals using e-cigarettes as a smoking cessation aid are more likely to desire stronger throat hit than non-smokers that are new to e-cigarettes (McQueen et al., 2011; Barbeau et al., 2013; Li et al., 2016). It is important to keep this distinction in mind since many human studies employ adult smokers or dual users who may prefer a greater throat hit.

### Unprotonated (Free-Base) Versus Protonated Nicotine

The sensory properties of aerosolized nicotine delivered from e-cigarettes are altered by the pH of the carrier e-liquid. Nicotine can exist either as unprotonated (*i.e.*, free-base) or protonated (*i.e.*, mono- or di-protonated). Since nicotine is a weak base with a pKa of 8.02, a higher pH results in a greater proportion of unprotonated, free-base nicotine. Importantly, free-base nicotine is also more volatile (Pankow, 2001). Therefore, more basic nicotine formulations result in an increased proportion of nicotine in the gaseous state upon heating and aerosolization, leading to greater deposition and subsequent absorption of nicotine in the mouth and upper airway (Pankow, 2001; Hukkanen et al., 2005). The tradeoff, however, is greater irritation and perception of bitterness (Caldwell et al., 2012; Voos et al., 2019). In contrast, there is a greater proportion of less volatile, protonated nicotine at a more acidic pH. Upon heating, more nicotine is present in the aerosol as a liquid, and the particulate matter deposits more effectively in the lungs with reduced harshness and a smoother taste (Pankow, 2001; Hukkanen et al., 2005). The smoke from most cigarettes, such as the popular Marlboro brand, tends toward weakly acidic while cigar smoke is more basic.

It may be assumed that protonated nicotine results in the greatest amount of systemic nicotine exposure due to its deposition in the lungs and its more favorable sensory profile. However, an important consideration is that nicotine can only pass through biological membranes in its unprotonated form – protonated nicotine must be physiologically buffered before it can be absorbed into the bloodstream (Pankow et al., 1997; Pankow, 2001; Hukkanen et al., 2005). Therefore, more acidic nicotine may result in less overall bioavailability. Basic nicotine, however, while absorbed more slowly in the mouth, would be more bioavailable. In this section we discuss the sensory implications of pH on e-cigarette use and the impact of nicotine and characterizing flavors. We do note that there is some debate as to how drastically nicotine pH alters pharmacokinetic parameters, and evidence exists that nicotine absorption and bioavailability are not affected at a pH ≤ 8 (Dixon et al., 2000; Seeman, 2007; Klus et al., 2012; Shao et al., 2013).

The pH of e-liquids varies widely, ranging anywhere from ∼4 to ∼9, depending on factors such as nicotine content and the presence of various additives (Brunnemann and Hoffmann, 1974; Lisko et al., 2015; St.Helen et al., 2017). Because of nicotine’s alkalinity, an e-liquid’s pH usually correlates with total nicotine concentration – a higher concentration results in a more basic e-liquid. However, when examining a range of flavored commercial products, the relationship weakens due to the individual properties of the additives (Lisko et al., 2015). For example, a blueberry e-liquid with a nicotine content of 3.7 mg/mL has a pH of 7.3 while a watermelon flavored e-liquid with a nicotine content of 3.3 mg/mL has a pH of 7.7 (Lisko et al., 2015). Based on higher nicotine content alone, one would expect the blueberry e-liquid to be more basic, however, properties of the flavor additives impact the projected pH.

As previously mentioned, more acidic nicotine formulations have a smoother, less irritating sensory profile, which may alter user behavior, including puff topography. One study examining puff topography across different e-cigarette additives found that vaping behavior did in fact change between a strawberry- vs tobacco-flavored e-cigarette, as individuals took longer puffs from the strawberry e-cigarette, although the total number of puffs did not vary (St.Helen et al., 2018). As a possible explanation for this effect, the authors noted that the strawberry e-liquid was more acidic (pH 8.29) than the tobacco-flavored e-liquid (pH 9.10), which may alter nicotine absorption. An alternative explanation may be that a more acidic e-liquid results in a less irritating sensory profile, which could facilitate longer draws from an e-cigarette. However, it is difficult to isolate the effects of pH on sensory irritation due to differences in flavor or nicotine concentration between e-liquids. Future studies may consider evaluating ratings of harshness and irritation as a function of pH.

Tobacco companies dedicated years of research investigating how pH impacts cigarette preferences (Chen, 1976; Duell et al., 2020). For example, levulinic acid was commonly added to cigarettes to increase nicotine yields while maintaining a smoother sensory profile (Keithly et al., 2005). Some popular e-cigarette brands have leveraged this early research to patent their own nicotine formulations, nicotine salts, which have becoming increasingly prevalent in recent years (Bowen and Xing, 2015; Duell et al., 2020; Pennings et al., 2022). Nicotine, when conjugated to an acid such as salicylic, benzoic, or levulinic acid, yields nicotine concentrations ≥50 mg/mL at a pH of ∼6 (Fadus et al., 2019; Harvanko et al., 2020). Therefore, even with record-high nicotine concentrations, nicotine salts maintain a smoother, less irritating sensory profile. The more appealing sensory profile may contribute to a less aversive first-time experience, facilitating the use of a product with alarmingly high nicotine concentrations. In support of nicotine salt’s less irritating sensory profile, salt-based e-liquids (pH 6.6) are rated as sweeter, smoother, less bitter, and less harsh than free-base formulations (pH 8.9) with matched nicotine concentrations (Leventhal et al., 2021). Furthermore, sweetness and smoothness are positively associated with appeal while bitterness and harshness are negatively associated with appeal (Leventhal et al., 2021; Pang et al., 2021). An important consideration in comparisons of free-base and protonated nicotine e-liquids is that protonation status itself does not affect overall nicotine yield emitted from an e-cigarette when controlling for device power and levels of propylene glycol (PG) (Talih et al., 2020). This suggests that the improved sensory profile is dependent on pH-mediated effects rather than differences in nicotine yield and subsequent irritation. Ultimately, more research is needed in order to adequately examine the impact of nicotine formulation on sensory perception and e-cigarette use.

## E-Liquid Components

### Propylene Glycol and Vegetable Glycerin

Along with nicotine and various flavoring compounds, e-liquids are primarily comprised of PG and vegetable glycerin (VG). PG and VG are effective humectants and solvents, carrying flavor while simulating cigarette smoke upon heating and aerosolization. PG, VG, and many common flavor additives are “generally recognized as safe” by the US FDA; however, this label applies to substances used as food additives rather than inhalants (Sears et al., 2017). The toxicology of these products when heated and inhaled has become a growing concern (for review, see Traboulsi et al., 2020).

Propylene Glycol, a slightly viscous, clear liquid has virtually no odor but is sometimes described as tasting slightly sweet. Across social media platforms and various forums, e-cigarette users report both higher nicotine levels and higher PG ratios as producing greater throat hit (Li et al., 2016). While nicotine’s irritant effects are well-established, PG may have irritant effects of its own. For example, exposure to PG-based vapor can cause acute eye and airway irritation, along with chronic respiratory dysfunction (Moline et al., 2000; Wieslander et al., 2001; Varughese et al., 2005). Furthermore, PG, sometimes used as a vehicle solution for intravenous drug delivery, can induce burning or stinging sensations during injection (Doenicke et al., 1999). As a possible explanation of its nociceptive effects, PG activates heterologously expressed human TRPA1 and rat TRPV1 receptors (Niedermirtl et al., 2018). However, the interaction between PG and TRPA1 in e-cigarette users is currently unknown.

PG also alters nicotine emissions from e-cigarette devices. Since PG is more volatile than VG and has a greater vaporization rate, increasing PG concentration increases the rate at which nicotine is emitted (Talih et al., 2017). Furthermore, higher PG content results in greater nicotine emissions, at a magnitude of 4× in some conditions (Baassiri et al., 2017). Together, these studies highlight the importance of PG content in overall nicotine delivery, even when nicotine concentrations are held constant. Ultimately, a higher ratio of PG to VG can increase nicotine emissions, which can exacerbate overall irritation.

VG, or glycerol, is a syrupy liquid with a sweet taste quality. Compared to PG, VG-based e-liquids produce a larger, longer-lasting exhaled aerosol due to its greater particle size and slower evaporation time (Baassiri et al., 2017; Harvanko et al., 2019; Vena et al., 2019). Some users, often termed “cloud-chasers,” balance their PG:VG ratio to favor VG in order to obtain a better cloud (Tokle and Pedersen, 2019), highlighting the role of visual sensory input on use. Similar to that of other sensory cues, visual clouds may evolve into conditioned reinforcers; clouds emitted from high VG-containing e-cigarettes induce greater urge and desire to smoke than clouds emitted from a low VG e-cigarette (Vena et al., 2019).

Because of its role as a thickening agent, liquid cough medicines often use VG. However, some evidence points to VG’s demulcent, lubricant, and sweet properties as actual contributors to cough suppression (Eccles and Mallefet, 2017). Interestingly, rinsing the mouth with sucrose prior to inhaling a respiratory irritant decreases cough reflex sensitivity (Wise et al., 2012). Since VG is about 60–80% as sweet as sucrose (Eccles and Mallefet, 2017), it may have similar functions. Ratings of sweetness are a common parameter evaluated in e-cigarette studies. As will be discussed later, many popular flavor additives such as cherry are rated as sweet, and sweetness is positively associated with liking (Baker et al., 2021a). However, the role of VG ratio on ratings of sweetness and association with liking has not been examined. In sum, higher VG content in e-cigarettes may help mitigate irritation and cough reflex induced by nicotine and PG while adding sweetness, thus contributing to a more pleasant experience. This may be particularly important for first time users who are unaccustomed to the sensory irritation induced by nicotine.

Between PG:VG ratio and varying device types, e-cigarettes allow users a customizable experience. E-cigarette product websites, user blogs, and online forums describe different ratios to use in order to achieve one’s desired sensory appeal (*e.g.*, optimal throat hit, cloud production, taste, *etc*.). Interestingly, however, some studies have challenged the importance of PG:VG ratio in user experience. Experienced e-cigarette users were asked to determine if they could differentiate between watermelon-flavored, nicotine-containing e-cigarettes of different PG:VG ratios. Even with the most drastic comparisons between 30:70 and 70:30 ratios, only ∼33% of the 14 participants could detect a difference (Schneller et al., 2018). Furthermore, when participants who had never used e-cigarettes before were asked to compare e-cigarettes with different PG:VG ratios, they did not rate satisfaction, enjoyment, or cloud production differently (Smith et al., 2020), further questioning the impact of PG:VG ratio on subjective experience. It is important to note that these studies used specific nicotine concentrations and device types, factors which can have a large influence on sensory experience and aerosol generation.

## Characterizing Flavors Have Distinct Sensory Effects

### Menthol

Menthol, a naturally occurring, aromatic alcohol found in mint plants and some essential oils, is a common additive in both consumer and tobacco products (Berg, 2016; Leventhal et al., 2019), and menthol-containing e-cigarettes are some of the most popular across age groups (Nguyen et al., 2019; Krüsemann et al., 2021). Menthol can exist as different stereoisomers; however, naturally occurring menthol is largely (>98%) L-menthol with small amounts of D-menthol and other isomers (Eccles, 1994). Menthol has direct pharmacological actions in the central nervous system that may impact nicotine use, such as altering ventral tegmental area dopamine neuron excitability, upregulating nAChRs, slowing nicotine metabolism, and becoming a predictive, conditioned cue for nicotine reward (for review, see Wickham, 2021). In behavioral assays, some studies have found that menthol, delivered intraperitoneally, impacts nicotine reward and seeking (Biswas et al., 2016; Harrison et al., 2017; Henderson et al., 2017), while others argue that menthol contrastingly inhibits nicotine’s reinforcing effects (Nesil et al., 2019). Several factors may account for these differences in results, such as route and timing of menthol administration and differences in behavioral paradigms. For the purpose of this review, however, we will focus on literature surrounding menthol’s pre-ingestive, sensory effects and possible implications for e-cigarette use.

Menthol is probably most well-known for its characteristic cooling effects. The cooling properties of menthol are mediated through activation of TRP melastatin 8 (TRPM8) receptors expressed throughout the mouth, nose, throat, and lungs (McKemy et al., 2002; Wickham, 2021). TRPM8, also known as cold and menthol receptor 1 (CMR1), is activated by chemical agents such as menthol or by temperatures below 26°C (Bautista et al., 2007).

Menthol can be found in various medicines and lozenges due to its antitussive properties. For example, inhalation of menthol vapor increases the concentration of inhaled capsaicin needed to elicit cough (Wise et al., 2012), an effect thought to rely upon TRPM8 receptors expressed in nasal trigeminal afferent neurons (Plevkova et al., 2013). Menthol can also act as an anti-irritant and analgesic. While some evidence exists that menthol’s analgesic effects are centrally-mediated (Galeotti et al., 2002; Watt et al., 2008; Zhang et al., 2008), other studies point to its peripheral actions. Menthol can inhibit Ca^2+^ and Na^+^ channels on sensory neurons, thus dampening nociception (Swandulla et al., 1987; Gaudioso et al., 2012). However, Liu et al. (2013), utilizing knockout mouse models and various routes of menthol administration (*i.e.*, oral, intraperitoneal, intraplantar, and topical) found that menthol’s analgesic effects are almost exclusively dependent on TRPM8. These discrepancies may stem from use of different methodology or use of menthol isomers. For example, the work of Gaudioso et al. on inhibition of Na^+^ channels tested D-menthol while Liu et al. used L-menthol. In sum, menthol’s cooling, antitussive, anti-irritant, and analgesic effects may alter nicotine’s perceived sensory profile and reduce irritation experienced during e-cigarette use. This is especially important in new users, since a more pleasurable first time experience with tobacco products is predictive of future use (de Wit and Phillips, 2012).

While having an innocuous cooling sensation, menthol also induces oral and nasal irritation (Klein et al., 2011). Sub- to low-micromolar concentrations of menthol activate heterologously expressed mouse TRPA1 receptors, while higher concentrations block TRPA1 (Karashima et al., 2007). However, in heterologously expressed human TRPA1 receptors, menthol acts as an agonist at low and high concentrations (Xiao et al., 2008), a phenomenon since replicated by other groups which likely contributes to menthol itself as an irritant. While relevant, these effects may desensitize rapidly. In human subjects, menthol applied to the tongue elicits a self-desensitization effect, where a repeated presentation, even 60 mins later, induces far less irritation (Klein et al., 2011). Menthol also reduces the irritating effects of cinnamaldehyde, a TRPA1 agonist (Klein et al., 2011). Therefore, while menthol may have initial irritant effects, subsequent and prolonged use can decrease irritation of itself and other compounds. One human study found that menthol vapor inhaled nasally decreases irritation of inhaled acetic acid, a weak activator of TRPA1 and acid-sensing ion channels (Wang et al., 2011; Wise et al., 2011; Nielsen, 2018). Interestingly, however, the same study found that menthol pretreatment increases nasal sensitivity to allyl isothiocyanate (AITC), a potent TRPA1 agonist (Wise et al., 2011). While this finding seems at odds with the effects of acetic acid and previously discussed work on oral irritancy, differences may arise due to AITC’s potency, route of administration, or time between menthol/irritant presentations. For example, Klein et al. administered cinnamaldehyde 5, 30, or 60 mins after applying menthol to the tongue, while Wise et al. applied menthol and the TRPA1 agonist within seconds from each other. Previous studies have found that consecutive inhalations of AITC with an inter-stimulus interval of less than 2 mins increases irritation intensity, yet when delivered three or more minutes apart reduces irritation intensity (Brand, 2002). While this indicates a self-desensitization effect, it is likely applicable to cross-desensitization effects as well and may explain variations in results. Inter-stimulus interval is an important consideration when interpreting these studies and drawing parallels to e-cigarettes, since menthol and various irritants (*i.e.*, nicotine) are presented simultaneously, rather than successively, when vaping. While e-cigarette users vary widely in how they vape (Behar et al., 2015; Lee Y. O. et al., 2018; Patten and De Biasi, 2020), they take multiple puffs during a single vape session and engage in multiple sessions each day. Therefore, it is likely that menthol’s self-desensitization and cross-desensitization effects are most relevant beyond the initial puff and may make nicotine use more tolerable and less irritating over the course of a single vape session or an entire day.

Menthol is “promiscuous” in its actions with sensory channels, interacting not only with TRPM8 and TRPA1, but also with nAChRs, TRPV1, and TRPV3 (Macpherson et al., 2006). As previously discussed, nicotine’s irritant effects are predominately mediated by nAChRs. Some studies have found that menthol acts as a negative allosteric modulator of nAChRs expressed in sensory neurons, which may therefore alter nicotine’s sensory effects (Hans et al., 2012; Ton et al., 2015). Furthermore, some of menthol’s counter-irritant effects could also be explained by its interactions with TRPV1 channels, as menthol decreases TRPV1 currents activated *via* heat and capsaicin (Takaishi et al., 2016). Menthol’s actions on TRPV3 receptors and subsequent implications for nicotine use are unknown. TRPV3 is highly expressed in keratinocytes and around hair follicles, with some expression in other regions such as the brain, tongue, and larynx (Yang and Zhu, 2014). Compared to other TRP channels, expression of TRPV3 in sensory neurons is minimal (Yoshioka et al., 2009).

When directly examining nicotine consumption (see Table 2), male mice prefer mentholated nicotine solutions in a two-bottle choice task, a result that is lost in TRPM8-KO mice (Fan et al., 2016). Interestingly, Wickham et al. (2018) also found that male rats increase oral nicotine consumption when menthol is present, but do not increase lever pressing for intravenous nicotine delivery with concurrent oral menthol delivery. This study supports the notion that menthol enhances nicotine intake by altering its palatability. Conversely, work performed in female rats reported increased responding for intravenous nicotine delivery with a concurrent intraoral menthol compared to intraoral vehicle (Wang et al., 2014). These results suggest that menthol’s characteristic sensory effects may serve as a conditioned cue when paired with nicotine delivery. However, in the latter study, rats obtained nicotine + menthol deliveries through licking on an active spout rather than lever pressing; therefore, it is difficult to make parallels between studies. Furthermore, neither study examined sex differences, an important consideration since in humans, women are more likely to use mentholated cigarettes, and similar trends have emerged for menthol-containing e-cigarettes (Smith et al., 2017; Pang et al., 2020). Bagdas et al. (2020), using a two-bottle choice task to examine nicotine consumption in male and female rats, found that only males show an increased preference for menthol-containing solutions. This finding is consistent with previous reports showing that male, but not female, mice increase oral nicotine consumption when menthol is added to the drinking solution (Fait et al., 2017). In a follow up study, orofacial movements were examined in response to varying concentrations of nicotine with or without added menthol delivered *via* intraoral catheters. Following nicotine’s inverted U-shaped dose-response curve, this study found that menthol increases hedonic responses (*i.e.*, tongue protrusions, rhythmic mouth movements) to low nicotine concentrations and decreases aversive responses (*i.e.*, head shakes, forelimb flails, and gaping) to high nicotine concentrations in males (Bagdas et al., 2021). In perhaps a more translatable method, the effects of menthol vapor on respiratory responses to inhaled irritants have also been examined. In female mice, menthol reduces respiratory irritation in response to acrolein, a TRPA1 agonist, and acetic acid and cyclohexanone, TRPV1 agonists, all of which are irritants found in cigarette smoke (Willis et al., 2011; Ha et al., 2015). While acetic acid and cyclohexanone are unique to cigarette smoke, e-cigarette aerosol does contain acrolein along with a variety of other components which activate TRPV1 and TRPA1 (Ogunwale et al., 2017; Niedermirtl et al., 2018; Cunningham et al., 2020). It is possible that orosensory experience may account for variations in responses between male and female rodents, or that a broader range of menthol concentrations are needed to fully tease apart effects in female rodents. Furthermore, the balance between menthol’s pre-ingestive (*e.g.*, bitter taste responsivity, susceptibility to peripheral irritancy) and post-ingestive (*e.g.*, alterations in nicotine metabolism, nAChR expression) effects likely vary between sex in both humans and rodents.

**TABLE 2:** Summary of menthol’s effects on nicotine use.

Species	Route	Doses	Nicotine pH	Main findings	References
C57BL/6 mice, adult males	Oral	Menthol (10–200 μg/ml) Nicotine (50–200 μg/ml)	“Base”	• Male mice prefer mentholated nicotine solutions (50 μg/ml menthol, 200 μg/ml nicotine) over nicotine alone in two-bottle choice task	Fan et al., 2016
Sprague Dawley rats, adult males	Oral, intravenous (i.v.)	Menthol (0.005% w/v) Nicotine (0–100 mg/L; 30 μg/kg/infusion)	*Oral-7.4 *i.v.- free-base	• Rats increase oral nicotine consumption (50, 100 mg/mL) when menthol present compared to nicotine alone in two-bottle choice task	Wickham et al., 2018
				• Rats do not increase lever pressing for i.v. nicotine delivery with concurrent intraoral menthol delivery	
Sprague Dawley rats, adolescent females	Oral, i.v.	Menthol (0.01% w/v) Nicotine (30 μg/kg/infusion)	7.0–7.4	• Rats increase responding (licking at active spout) for i.v. nicotine delivery with a concurrent intraoral menthol compared to i.v. nicotine with intraoral vehicle	Wang et al., 2014
Sprague Dawley rats, adult males and females	Oral	Menthol (100–1,000 mg/L) Nicotine (3, 20 mg/L)	Free-base	• Only male rats show an increased consumption of menthol-containing solutions in two-bottle choice task	Bagdas et al., 2020
C57BL/6J mice, adolescent and adult males and females	Oral	Menthol (10 μg/ml) Nicotine (200 μg/ml)	7	• Only adult male mice increase oral nicotine consumption when menthol is added to drinking solution	Fait et al., 2017
Sprague Dawley rats, adult males and females	Oral	Menthol (50 μg/ml) Nicotine (0–30 μg/ml)	7	• Menthol increases hedonic responses (tongue protrusions, rhythmic mouth movements) to nicotine (30 μg/ml) in males	Bagdas et al., 2021
				• Menthol decreases aversive responses (head shakes, forelimb flails, gaping) to nicotine (30 μg/ml) in males	
Human, N = 32, 50% male, age 18–45	Inhalation	Menthol (0–3.5%) Nicotine (0–24 mg/ml)	n/a	• Participants rate menthol-containing e-cigarettes (3.5% menthol, 24 mg/ml nicotine) as less harsh than nicotine-only e-cigarettes	Rosbrook and Green, 2016
Human, N = 32, 66% male, age 18–50	Inhalation	Menthol (3.5%) Nicotine (0, 24 mg/ml)	n/a	• Menthol reduces ratings of aversiveness and dislike of 24 mg/ml nicotine e-cigarettes	Devito et al., 2020
Human, N = 60, 48% male, age 16–20	Inhalation	Menthol (0–3.5%) Nicotine (0–12 mg/ml)	n/a	• A high concentration of menthol (3.5%) increases liking of 12 mg/ml nicotine e-cigarettes	Krishnan-Sarin et al., 2017
				• Low (0.5%) and high (3.5%) menthol concentrations improve ratings of 12 mg/ml nicotine e-cigarette taste	
Human, N = 49, 63% male, age 16–20	Inhalation	Menthol (n/a) Nicotine (6, 12 mg/ml)	Free-base	• Menthol has no effect on taste palatability	Jackson et al., 2021

*Reported nicotine pH differs between administration routes.

Menthol, when applied to the tongue, reduces nicotine’s irritating effects (Dessirier et al., 2001). Interestingly, however, menthol appears to have no effect on nicotine-induced nasal irritation (Renner and Schreiber, 2012). In the latter study, Renner et al. introduced menthol during the second half of a session after nicotine had already been presented 15 consecutive times. It is possible that nicotine’s pain-inducing effects had plateaued to a point where menthol would have no further ability to reduce irritation. In support of menthol’s anti-irritant, “masking” effects, and in direct examination of concurrent nicotine and menthol administration *via* e-cigarette, Rosbrook and Green (2016) found that participants rate menthol-containing e-cigarette solutions as less harsh than nicotine-only solutions. This effect, however, was only seen at a 3.5% menthol concentration and a higher nicotine concentration of 24 mg/mL nicotine (Rosbrook and Green, 2016). Devito et al. (2020) similarly found in adult smokers that 3.5% menthol reduces ratings of aversiveness and dislike at 24 mg/ml nicotine concentration. Importantly, participants in both studies were almost all menthol cigarette smokers, which may ultimately impact their overall perception and experience with menthol. While these studies employed adult cigarette smokers (ages 18–45/50), other studies conducted in young e-cigarette users (ages 16–20), only ∼50% of whom were menthol users, found that menthol increases overall ratings of e-cigarette liking and improves ratings of taste in a 12 mg/mL nicotine condition (Krishnan-Sarin et al., 2017). However, a follow-up study found that menthol has no effect on taste palatability in 16–20 year old e-cigarette users (Jackson et al., 2021). An important difference between the two studies were the PG:VG ratios, as the first used 70:30 PG:VG and the latter 50:50 PG:VG. While the implications of this are discussed at length previously, the difference in PG:VG ratio may have altered total nicotine emissions and nicotine-induced irritation, thus contributing the menthol’s effects or lack thereof. While the work of Krishnan-Sarin et al. did not make direct comparisons of harshness and irritation to investigate a masking effect, it does support menthol’s ability to improve palatability in certain circumstances. One such circumstance is among individuals with heightened sensitivity to bitterness (*e.g.*, PROP tasters). Among pregnant smokers, women sensitive to PROP bitterness are more likely to smoke menthol cigarettes (Oncken et al., 2015). Another study examining both men and women found a similar relationship between menthol smoking status and PROP sensitivity (Duffy et al., 2019). Even among e-cigarette users, PROP tasters are more likely to prefer menthol-flavored than unflavored e-cigarettes (Mead et al., 2019). Together, these studies suggest that smokers with a higher propensity for bitter taste combat nicotine’s bitter and aversive effects through use of mentholated products.

In sum, menthol may promote and perhaps escalate nicotine use due to its widespread effects on sensory perception. A study of high school students who vaped cooling flavors such as menthol found that the use of cooling flavors is associated with more frequent vaping and an increased likelihood of vaping nicotine-containing products (Davis et al., 2021). This further supports the idea that menthol facilitates nicotine use, perhaps due to its counter-irritant or masking effects. Studies that have identified a link between bitter taste perception and menthol similarly support menthol’s masking ability. Finally, the widespread use of menthol in consumer products, some of which are used daily (*e.g.*, toothpaste or mouthwash), could contributed to familiarity with menthol that may add desirability, further supporting its use in e-cigarettes.

### Impact of Other Characterizing Flavors on Nicotine Use

Of all the characterizing flavors, menthol has, by far, received the most attention. However, less is known about the sensory impact other flavor additives have on nicotine use. Along with mint and menthol, fruit flavors are most popular across all age groups (Harrell et al., 2017; Nguyen et al., 2019; Groom et al., 2020; Gravely et al., 2021). Current research suggests that various additives, in a similar fashion as menthol, may have direct pharmacological actions. For example, farnesol and farnesene, chemical flavorants that contribute to a “green apple” flavor, trigger reward-related behaviors when injected intraperitoneally in rodents (Avelar et al., 2019; Cooper et al., 2020). There is great merit to investigating the direct actions of flavor additives alone, as a large proportion of e-cigarette users vape only flavor (Miech et al., 2017). Other research points to the ability of flavors to enhance nicotine reward and reinforcement in both humans and rodent models (for review, see Patten and De Biasi, 2020). Here we will focus on the sensory implications of flavor additives and their relation to nicotine intake and will therefore cover studies wherein flavors were administered orally or in vapor form (see Table 3).

**TABLE 3:** Summary of characterizing flavors (*sans* menthol) on nicotine use.

Flavor	Species	Route	Concentration/Dose	Nicotine pH	Main findings	References
Sucrose	Wistar rats, adult males	Oral	Sucrose (0–10%)	n/a	• Rats increase consumption of nicotine when solutions are sweetened with sucrose	Smith and Roberts, 1995
			Nicotine (10 μg/ml)			
“Vanilla,” “Coconut”	C57BL/6J mice, adult males	Oral	Vanilla, coconut (0.01–1%) Nicotine (40–120 μg/ml)	7.7–7.9	• Mice consume more vanilla-flavored (1%) nicotine solutions (60 mg/ml) than nicotine only solutions	Tannous et al., 2021
					• Vanilla flavor enhances oral nicotine self-administration compared to nicotine alone	
“Retro Fruit Twist,” “Tobacco”	C57BL/6J mice, adult males	Oral	Flavors (n/a) Nicotine (30–200 μg/ml)	Free-base	• Mice consume more fruit-flavored nicotine solutions than nicotine-only solutions (75, 100, and 200 mg/ml)	Wong et al., 2020
					• Mice do not show increased consumption of tobacco-flavored nicotine solutions compared to nicotine-only solutions	
“Strawberry”	C57BL/6J mice, adolescent-adult males and females	Oral	Strawberry (Unsweetened Strawberry Kool-Aid made in 2% saccharin)	Free-base	• Adolescent mice prefer strawberry-flavored nicotine solutions over nicotine-only solutions	Patten et al., 2021
			Nicotine (0.1 mg/ml)		• Adolescent females show greater preference than adolescent males in this effect	
“Chocolate,” “Grape”	Sprague Dawley rats, adolescent females	Oral, intravenous (i.v.)	Chocolate (0.5% Hershey’s Unsweetened Cocoa)	Free-base	• Rats do not self-administer i.v. nicotine with contingent intraoral flavor delivery	Chen et al., 2011
			Grape (0.1% Unsweetened Grape Kool-Aid)			
			Nicotine (15–30 μg/kg/infusion)			
			*0.4% saccharin added to oral solutions			
“Licorice”	Sprague Dawley rats, adult males	Oral, i.v.	Licorice (0.1, 1.0% vol/vol licorice root extract)	Free-base	• Licorice (1.0%) as a conditioned reinforcer prior to self-administration testing increases operating responding for nicotine infusions whereas unconditioned licorice does not	Palmatier et al., 2020
			Nicotine (7.5 μg/kg/infusion)			
Saccharin, sucrose	Sprague Dawley rats, adult males	Oral, i.v.	Saccharin (0.32%)	Free-base	• Contingent delivery of intraoral sucrose or saccharin enhances self-administration of i.v. nicotine obtained *via* lever pressing	Wickham et al., 2018
			Sucrose (10%)			
			Nicotine (0, 30 μg/kg/infusion)			
“Sweet flavors” – peach, watermelon, blackberry, cotton candy, cola, sweet lemon tea	Human, N = 20, 55% male, age 19–34)	Inhalation	Flavors (n/a) Nicotine (0, 6 mg/ml)	Free-base	• Sweet-flavored e-cigarettes increase appeal ratings compared to tobacco, menthol, and unflavored e-cigarettes	Goldenson et al., 2016
“Cherry Crush,” “Vivid Vanilla,” “Piña Colada,” “Peach Schnapps”	Human, N = 31, 58% male, average age = 34) *age range n/a	Inhalation	Flavor (n/a) Nicotine (12 mg/ml)	Free-base	• Piña Colada rated as sweetest and most liked • Sweetness is positively associated with liking • Harshness is negatively associated with liking	Kim et al., 2016
“Cherry,” “Chocolate”	Human, N = 132, 49% male, age 18–45)	Inhalation	Flavors (n/a) Nicotine (18 mg/ml)	Free-base	• Individuals rate cherry and chocolate e-cigarettes as sweeter than unflavored e-cigarettes, but not more liked	Mead et al., 2019
					• Sweetness is positively associated with liking	
					• Irritation and bitterness are negatively associated with liking	
“Cherry,” “Chocolate”	Human, N = 39, 100% male, age 18–45	Inhalation	Flavor (n/a) Nicotine (6, 18 mg/ml)	n/a	• Sweetness is positively associated with first puff liking	Baker et al., 2021a
					• Harshness/irritation is negatively associated with first puff liking	
					• First puff liking is not associated with total nicotine intake	
“Cherry”	Human, N = 19, 68% male, age 21–35)	Inhalation	Cherry (4.7% or 9.3% vol/vol) Nicotine (0, 6, 12 mg/ml)	Free-base	• Increasing nicotine concentration increases ratings of bitterness and reduces appeal	Pullicin et al., 2020
					• Cherry flavor increases ratings of sweetness and liking	
					• Increasing the concentration of cherry flavor from 4.7 to 9.3% increases perceived sweetness, harshness, and bitterness but does not alter hedonic ratings	

Early studies have found that sweetening nicotine solutions with sucrose increases consumption compared to nicotine-only solutions (Smith and Roberts, 1995). Regarding common e-cigarette flavor additives, the popular additive vanilla mitigates the natural aversion male mice display to oral nicotine solution and enhances nicotine consumption in a two-bottle choice task (Tannous et al., 2021). However, vanilla does not enhance operant responding for oral nicotine, suggesting that it does not impact nicotine’s reinforcing properties (Tannous et al., 2021). Similarly, adolescent and adult mice alike prefer fruit-flavored solutions over nicotine alone or tobacco-flavored solutions (Wong et al., 2020; Patten et al., 2021). Only one of these studies examined sex differences and found that while both sexes prefer a strawberry + nicotine solution over nicotine only, adolescent females tend to show greater preference than adolescent males in this effect (Patten et al., 2021). However, it is difficult to separate to what extent flavors act as conditioned reinforcers or as enhancers of nicotine’s palatability in these studies.

Chen et al. combined intravenous nicotine delivery with oral flavor additives consumed *via* licking. While adolescent female rats acquire stable nicotine self-administration, the addition of an appetitive olfactogustatory stimulus prevents nicotine self-administration, suggesting that rats instead form a conditioned taste aversion to nicotine (Chen et al., 2011). Wickham et al. (2018), however, found that contingent delivery of intraoral sucrose or saccharin enhances self-administration of intravenous nicotine obtained *via* lever press. The reinforcing value of sucrose alone did not change after pairing with nicotine, suggesting that the primary driver of enhanced nicotine self-administration is the appetitiveness of the cue (Wickham et al., 2018). Importantly, in this study rats were originally trained to lever press for intraoral sucrose delivery, which is argued as translationally relevant, since human e-cigarette users almost undoubtedly have previous, oftentimes positive experiences with sweetness before initiating nicotine use (Wickham et al., 2018). In line with the importance of previous experience, Palmatier et al. (2020) examined nicotine self-administration with concurrent delivery of an oral flavor cue. Prior to nicotine administration, licorice flavor was established as a conditioned reinforcer through pairing with sucrose in order to model previous experience with sweet flavors in humans. Licorice, as a conditioned reinforcer, increases operant responding for nicotine infusion while neutral licorice flavor does not (Palmatier et al., 2020).

A host of studies in humans started to investigate the chemosensory contributions of flavor additives on e-cigarette use. When liquid mixtures of PG:VG are delivered into the mouth along with simultaneous odorized air, different aromas have varying effects on ratings of sweetness, pleasantness, and bitterness, and even modulate the perceived taste of PG:VG; for example, fruity aromas increase ratings of PG:VG sweetness (Rao et al., 2018). Furthermore, simultaneous oral delivery of a bitter tastants with a sweet, “fruity,” aromatic flavorant, ethyl hexanoate, decreases ratings of bitterness (Isogai and Wise, 2016). Importantly, in either study, stimuli did not include nicotine and were not aerosolized *via* heating, two important features of human e-cigarette use. Nevertheless, these studies do provide proof-of-concept that popular flavor compounds can alter subjective sensory experience.

In studies directly examining e-cigarette use, sweet flavor additives such as piña colada, cotton candy, and cherry increase ratings of sweetness, and ratings of sweetness are positively associated with liking (Goldenson et al., 2016; Kim et al., 2016; Mead et al., 2019; Pullicin et al., 2020; Baker et al., 2021a). Conversely, subjects rate flavors such as tobacco as harsher, more bitter, and more irritating; these ratings are negatively correlated with liking (Kim et al., 2016; Mead et al., 2019; Baker et al., 2021a). A subset of these studies also found no evidence of a “masking effect” whereby flavors alter nicotine’s irritant properties. For example, harshness ratings of nicotine- containing e-cigarettes did not decrease with addition of cherry or other sweet flavors (Mead et al., 2019; Pullicin et al., 2020; Baker et al., 2021a). However, sweet flavor additives did improve overall ratings of liking as compared to nicotine-only e-cigarettes (Baker et al., 2021a). Therefore, it is possible that flavor additives, other than menthol, simply add a desirable, pleasant sensory experience rather than masking nicotine aversion. This is supported by work performed in young e-cigarette users age 16–20, where green apple containing e-cigarettes were rated as more liked but not less irritating (Jackson et al., 2021). It is important to note that the studies outlined here used concentrations of nicotine ranging from 6 to 18 mg/mL (Goldenson et al., 2016; Kim et al., 2016; Mead et al., 2019; Pullicin et al., 2020; Baker et al., 2021a). Menthol’s ability to reduce irritation and harshness is seen only at higher (24 mg/ml) nicotine concentrations and a 3.5% menthol concentration (Rosbrook and Green, 2016; Devito et al., 2020). Therefore, it is possible that a potential masking effect of sweet flavor additives would be more prevalent at higher nicotine concentrations or at different flavor additive concentrations. To examine this, Pullicin et al. (2020) tested a low and high concentration of cherry additive and found a trend for high cherry concentration to further increase liking, but simultaneously increase ratings of harshness. However, different flavor additives likely have different chemosensory effects, and due to the sheer number of available flavors, it is impossible for any one study to make widespread generalizations. Furthermore, the studies described within this section fail to evaluate nicotine salts, examining only free-base formulations. Considering the increasing prevalence and popularity of nicotine salts, especially among youth, further work is needed to better understand the sensory perception of flavored nicotine salt formulations and potential impact on e-cigarette use.

## Issues in Interpreting E-Cigarette Studies

There are a few important caveats to discuss when interpreting e-cigarette studies, some of which have been briefly touched upon. Firstly, e-cigarette emissions can vary across device type and company (El-Hellani et al., 2018). Aerosol generation and nicotine emissions are greatly impacted by the device power and liquid levels in the atomizer tank. Increasing power supplied to the heating element can increase the temperature of the aerosol, increasing overall vapor generation and the concentration of nicotine in the aerosol. Therefore, a high powered, low nicotine-containing e-cigarette may deliver more nicotine than a low powered, high nicotine device (Voos et al., 2019). Furthermore, e-liquid levels within a device can impact the temperature of the heating coils and influence vapor generation (Chen et al., 2018). Therefore, variations in emissions are likely, if not inevitable, between e-cigarette studies. Some available devices, such as e-cigarette “mods,” allow users to manually fill their e-cigarettes with liquid and adjust their power settings based on preference. This adds to the difficulty researchers have in recreating real-world settings. Another important consideration is the lack of regulation within the e-cigarette industry. Multiple studies have reported significant discrepancies between the labeled and actual nicotine content in e-liquids (Kim et al., 2015; Lisko et al., 2015; Raymond et al., 2018). Therefore, studies that use commercially available e-cigarette liquids without verification may lack control of true nicotine content. Taken together, it is difficult for e-cigarette studies to claim generalizability, simply based on the number of factors that can vary between users.

User age is another crucial consideration in e-cigarette use. As previously mentioned, flavored e-cigarettes are consistently rated as sweet, and sweetness is positively associated with liking (Goldenson et al., 2016; Kim et al., 2016; Mead et al., 2019; Pullicin et al., 2020; Baker et al., 2021a). Many e-liquids fail to list ingredients on the label, and those that do sometimes only list “sweeteners” rather than specific sugars (Fagan et al., 2018). However, sucrose, fructose, and glucose have all been identified in popular e-liquids (Kubica et al., 2014; Fagan et al., 2018), and many do-it-yourself vapers will add sugars to e-liquids as well (Patten and De Biasi, 2020). Therefore, both flavor volatiles and the inclusions of sweeteners can contribute to the overall enhancement of perceived sweetness in some e-cigarettes. Children and adolescents especially prefer high levels of sweetness, and this preference declines with age (Desor and Beauchamp, 1987; Petty et al., 2020). Therefore, from a chemosensory standpoint, adolescents may be particularly prone to experimentation and use of flavored products. Furthermore, sucrose can function as a cough suppressant, and may also play a role in reducing irritation during use (Wise et al., 2012). Due to ethical considerations, controlled studies in human adolescent users cannot be conducted. The studies outlined in this review were conducted in adult smokers or e-cigarette users over the age of 18, with only two that included individuals between 16 and 17 years old. Therefore, it is difficult to determine in a laboratory setting if differences arise between age groups.

## Future Considerations and Conclusion

Legislative restrictions against the use of characterizing flavors have already been put in place for combustible cigarettes, and measures are being taken against e-cigarettes as well. In February of 2020, the FDA banned the sale of reusable cartridges or pod-based e-cigarette products. However, disposable, flavored products containing characterizing flavors are still widely available. Furthermore, based on amendments to the Prevent All Cigarette Trafficking Act, the United States Postal Service, along with other major mail carriers, announced in 2021 they will no longer provide delivery of e-cigarette products direct to consumers. Despite these legislations, it is unlikely that e-cigarettes and characterizing flavors are going away. In one study on flavor preferences, ∼40% of participants reported they would “find a way” to obtain their preferred flavor in the advent of a flavor ban (Du et al., 2020). E-liquids, and even e-cigarettes themselves, can be made relatively easily with limited experience, ushering in a whole new era of “do-it-yourself” vaping. Therefore, while legislation may have some success in hindering the ease of youth acquisition, e-cigarettes will likely remain dominant players in the tobacco industry. As briefly mentioned, tobacco-free, synthetic nicotine products are quickly gaining popularity. Little is known, however, regarding the sensory effects of these products and how they compare to that of cigarettes or tobacco-derived e-cigarettes. Therefore, research into the role of flavor additives and the sensory aspect of vaping remains an important line of investigation, especially amidst the rise of new products.

## Author Contributions

NJ wrote the first draft of the review. All authors contributed to the revision and approved the final version for submission.

## Conflict of Interest

The authors declare that the research was conducted in the absence of any commercial or financial relationships that could be construed as a potential conflict of interest.

## Publisher’s Note

All claims expressed in this article are solely those of the authors and do not necessarily represent those of their affiliated organizations, or those of the publisher, the editors and the reviewers. Any product that may be evaluated in this article, or claim that may be made by its manufacturer, is not guaranteed or endorsed by the publisher.
